# Diabetic retinopathy: reversibility of epigenetic modifications and new therapeutic targets

**DOI:** 10.1186/s13578-017-0167-1

**Published:** 2017-08-15

**Authors:** Xinyuan Zhang, Lin Zhao, Brett Hambly, Shisan Bao, Kaiyue Wang

**Affiliations:** 1 Beijing Institute of Ophthalmology, Beijing Tongren Eye Center, Tongren Hospital, Beijing Ophthalmology & Visual Sciences Key Lab, Capital Medical University, Beijing, 100730 People’s Republic of China; 2 Charles Perkins Centre, The University of Sydney, Level 4 West, D17, Camperdown, NSW 2006 Australia

**Keywords:** Diabetic retinopathy, Gene, Epigenetic modifications, DNA methylation, Non-coding RNA, Chromatin remodeling, Histone modification, Therapeutic target

## Abstract

In recent years, considerable progress has been made in the molecular mechanisms of epigenetics in disease development and progression, the reversible characteristics of epigenetic modification provide new insights for the treatment of such diseases. The pathogenesis of diabetic retinopathy (DR) has not yet been fully understood, treatment of refractory and recurrent diabetic macular edema remains a big change in clinical practice. This review emphasizes that reversibility of epigenetic modification could provide a new strategy for the prevention and treatment of diseases.

## Background

The Human Genome Project found that the protein-coding DNA accounted for only 2% of the entire genome, and thus, proposed that some non-coding regions, such as capable of producing non-coding RNA, repeat fragments, and transposons, may also exercise certain functions. Moreover, the development of some diseases cannot be explained solely by the change in DNA sequence; other factors, including living environment, mental conditions, and stress may also play a vital role in the onset of diseases. Thus, the term epigenetics refers to heritable modifications that are not involved in the changes in the DNA sequence.

Epigenetics is also known as “prefix genetics,” “external genetics,” or “post-genetics,” indicating that in the absence of changes in the DNA sequence, the function of genes could undergo changes that are reversible and heritable. Occasionally, it may also refer to the studies on the process of physiological development [1]. Vital mechanisms of epigenetic modification include DNA methylation, histone acetylation, non-coding RNA regulation, and chromatin remodeling. These mechanisms are intrinsically involved in the development of various diseases including cancer, autoimmune diseases, neurodegenerative diseases, psychiatric diseases, and addictive diseases [2]. In addition to systemic diseases, other ophthalmic diseases such as dry eye, keratitis, uveitis, glaucoma, myopia, and fundus neovascularization may also be associated with epigenetic modifications. Recently, DR, the leading cause of blindness among the working population, has attracted particular attention, due to its high rate of causing blindness, increasing incidence, and significance in public health.

The four mechanisms of epigenetic modification are discussed below.

### DNA methylation

DNA methylation is the earliest discovered mechanism in epigenetic modification. It refers to the addition of methyl groups of S-adenosylmethionine (SAM) to DNA molecules catalyzed by DNA methyl transferase (DNMT). In mammalian cells, CpG exists mainly in two forms: one evenly distributed throughout the DNA sequences (60–90% are methylated), and the other grouped in clusters known as the CpG islands (generally protected and remain unmethylated). In the eukaryotic cells, CpG islands are often found in the regulatory regions of coding genes and are involved in gene expression and chromatin structure modification [2]. Hitherto, five enzymes have been identified in the DNMT family, namely DNMT1, DNMT2, DNMT3a, DNMT3b, and DNMT3L; however, among them, only DNMT1, DNMT3a, and DNMT3b are active for DNA methylation. DNA methylation deactivates the target genes or causes conformational changes in DNA, thereby affecting the protein-DNA interaction. In a human body, there are three states of DNA methylation: persistent hypomethylation, induced demethylation, and hypermethylation. The states of methylation are closely related to disease development; persistent hypomethylation and hypermethylation are found in some cancer cells.

### Histone modification

Histone modifications refer to methylation, acetylation, phosphorylation, adenylation, ubiquitination, ADP-ribosylation, and other modifications of histones in the presence of related enzymes. Histones are an integral part of epigenetics. The core histones (H2A, H2B, H3, and H4) could form a nucleosome with two H2A–H2B dimers and one H3–H4 tetramer. The histone core is globular, and only the N-terminal protein tails are unstructured [2]. Histone H1 is the linker histone but does not make up the nucleosome beads. It rather binds to the linker DNA (DNA that distinguishes the two histone complexes), and fixes the position of the nucleosome for the entry and exit of DNA [3]. Histone modification affects the transcriptional activity of genes, thereby playing a significant role in regulating DNA transcription, repair, and replication, as well as, alternative splicing and chromosome condensation [2].

### Non-coding RNA

Non-coding RNA refers to RNA molecules that do not encode proteins. These RNAs include those with known functions, such as rRNA, tRNA, snRNA, snoRNA, microRNA (miRNA), and circular RNA and others with unknown functions. miRNA is a single strand RNA containing 21–25 nucleotides that is involved in post-transcriptional regulation. It can bind to the 3′-UTR of target sequences and modulate the activity of mRNA, mainly by degrading the RNA or suppressing the protein translation [4, 5]. In humans, this process affects 1/3rd of the entire genome, and in mammals, it regulates the expression of >60% proteins. Furthermore, miRNA also participates in various pathological processes, including the inhibition of translation initiation, inhibition of translation, as well as, the regulation of mRNA deadenylation and degradation. Each miRNA has multiple target genes, and the same gene can be regulated by different miRNAs. Since the inhibition of protein translation is a critical regulation pathway, the complex mechanism of non-coding RNA realizes the fine regulation of gene expression.

### Chromatin remodeling

Chromatin remodeling refers to epigenetic mechanisms based on altered chromatin conformation. Since the nucleosome is the basic structural unit of chromatin, the remodeling of chromatin primarily involves structural or molecular changes in nucleosomes, histones and DNAs during the expression, replication, and recombination of genes. Chromatin remodeling can affect DNA methylation, DNA replication, recombination, and repair as well as gene expression. A previous study showed that, during the embryonic and fetal period, chromatin remodeling could encode “molecular memories” of diseases that a susceptible individual may later suffer in adulthood. A typical example is a cross-sectional study of a large population, which found that prenatal malnutrition and low body weight were associated with the metabolic X syndrome (type II diabetes, cardiovascular disease, and hypertension). The underlying mechanism could be elucidated as such: instead of DNA changes, chromatin remodeling during the embryonic or fetal period may affect the normal development and metabolism in organs [6].

Although different modification mechanisms affect specific epigenetic phenomena independently, they interact with each other and together determine the complex physiological processes. For example, the regulation of DNA methylation and miRNA modification requires the participation of DNTMs; the function of DNTMs, in turn, relies on histone modification, such as H3K9 methylation or histone acetylation. Thus, the inhibition of DNMTS would lead to H3K9 demethylation, and subsequently, the release of miRNA [7].

## Advancements in DR epigenetics

Previous studies suggested that the four mechanisms of epigenetic modification play significant roles in DR development. The most typical example was hyperglycemia, considered as a major determinant of the progression of DR. However, despite the well-controlled blood glucose in advanced DM patients, the development or progression of DR has been shown to not be alleviated or improved. This indicated that the exposure to hyperglycemia resulted in a “metabolic memory phenomenon”, wherein the epigenetic modifications played essential roles [8]. In the following section, the significance of the four epigenetic mechanisms in DR development will be elucidated further.

### DNA methylation

Previous studies suggest a positive association between DNA methylation and DR development; however, different forms of diabetes may involve various CpG islands to be methylated during the development of DR. Maghbooli et al. studied the content of 5-methylcytosine in leukocytes sampled from the peripheral blood of type II diabetes patients and found that patients with DR showed a significantly higher level of DNA methylation as compared to those without DR. This phenomenon indicated that higher DNA methylation level could be a potential risk factor for DR development. In addition, the study found that with the progression of DR, the level of systemic DNA methylation did not continue to increase in DR patients, suggesting that it occurred only in the early stages of diabetes [9]. Agardh et al. performed a genome-wide analysis of blood DNA methylation in 28 patients with proliferative DR (PDR) and 30 healthy controls. The study identified a total of 349 methylated CpG sites within 233 genes, including *TNF*, *CHI3L1* (*YKL*-*40*), *CHN2*, *GIRP*, *GLRA1*, *GPX1*, *AHRR*, and *BCOR*. Most of the CpG islands in PDR patients showed a decreased level of DNA methylation, whereas 28 CpG sites within 17 genes (including *AHRR*, *GIRP*, *GLRA1*, and *BCOR*) showed increased methylation (*P* < 0.05). Notably, the genes involved in the natural killer (NK) cell-mediated toxicity pathway showed a significantly higher level of methylation (*P* = 0.006) [10], indicating that methylation in peripheral blood cells could be used as a predictor for type I diabetes-complicated PDR.

Studies on animal models also suggested that retinal cells under hyperglycemia exhibited an altered level of CpG methylation as compared to normal cells. Tewari et al. analyzed DNA methylation in the retinal tissue of streptozotocin (STZ)-induced diabetic rats with stringently regulated (glycosylated blood glucose 6%) and less controlled blood glucose (glycosylated blood glucose 11%). The study found that when a rat was administered less controlled blood glucose for 3 months followed by another 3 months of strictly controlled blood glucose, the level of polymerase γ-1 (POLG1), an enzyme that participates in the process of prokaryotic DNA replication. continued to decrease, the D-loop was damaged, and the CpG islands located within the regulatory region of POLG were hypermethylated. Further studies also found that the hypermethylated CpG islands in POLG affected its binding with mitochondrial RNA and inhibited the transcriptional activity [11]. This phenomenon was also observed in retinal endovascular cells that were exposed to hyperglycemia, indicating that DNA methylation plays a major role in metabolic memory [12]. An in vitro study revealed that methylation and activation of the matrix metalloproteinase 9 (*MMP* 9) gene are significantly associated with DR [12]; activated MMP-9 could cause mitochondrial damage and accelerate the apoptosis of retinal vascular endothelia, thereby inducing DR development and progression. Studies performed in human cadaver eyes confirmed that hyperglycemia caused H3K9 acetylation in MMP-9 (Ac-H3K9), and p65 activation resulted in CpG methylation within MMP-9. Both the changes increased the MMP-9 expression and further aggregated the mitochondrial damage. Therefore, a potential therapeutic target may be to regulate methylation and demethylation of MMP-9, thus reducing mitochondrial damage, and DR could be prevented and controlled [13] (Table 1).

**Table 1:** The role of DNA methylation in the pathogenesis of diabetic retinopathy

Refs	Study type	DNA methylation
Diabetes Metab [9]	Clinical study (serum)	Global DNA methylation↑
BMC Med [10]	Clinical study (blood)	DNA methylation↓ in PDR, differential DNA methylation of 28 CpG sites in 17 genes (AHRR, GIPR, GLRA1, and BCOR)
Invest Ophthalmol Vis Sci [11]	Invitro/animal (STZ diabetic mice) Retina	DNA methylation of polymerase gamma (*POLG1*) gene ↑
Lab Invest [13] Diabetes [12]	Invitro/animal (STZ diabetic mice) Retina	Dnmt1 binding to Matrix metalloproteinase -9 promoter→MMP↑→5mC↓
Invest Ophthalmol Vis Sci [14]	Invitro/animal study (Bovine retinal endothelial)	mtDNA methylation↑

### Histone modification

Diabetic retinopathy is a neurovascular disease caused by hyperglycemia. Histone modification participates in neuronal apoptosis and vascular leakage, thereby facilitating DR development and progression [15, 16]. Histone methylation and acetylation are the two major mechanisms by which histone modification affects DR development.

#### Histone methylation

Histone lysine methylation (HLM) plays a vital role in various physiological processes such as gene expression, gene transcription (activation and inhibition), genomic stability [17], stem cell pluripotency [18], tumorigenesis [19] and inflammatory reaction [20]. The methyl transferase SUV39H1 methylates the lysine of histone H3 at the N-terminus, resulting in H3K9me3 [21]. The methyl transferase encoded by SUV39H2 has been demonstrated to lead to H3K9 methylation, resulting in the onset of DR. A recent cohort study comprising of 3000 DM patients also demonstrated that a polymorphism in the *SUV39H2* gene was significantly associated with microvascular complications [22]. Since the EZH2- and G9A-mediated epigenetic regulation of HLM is speculated to play a key role in delaying retinal neuronal degradation, it might represent a promising therapeutic target in the future treatment and prevention of DR.

#### Histone acetylation

The level of histone acetylation was significantly increased in the retina of rats with diabetes. Hyperglycemia caused H3 hyperacetylation, and the inflammatory cytokines, such as TNF and Cox, were significantly increased in cultured Muller cells. However, these changes could be reversed by the inhibitors of histone acetyltransferase [23]. Histone acetylation is associated with the activation of the NF-κB-dependent inflammation signaling pathway. Also, an imbalanced histone acetylation would result in the “metabolic memory phenomenon”, which mainly manifests as hypoacetylation in the promoter region of protective genes and hyperacetylation in the promoter region of pathogenic genes. Zhong et al. found that in STZ-induced diabetic rats, the transcription activity of HDAC1/2/8 (histone deacetylase) was increased in retinal endovascular cells, whereas the activity of HAT (histone acetyltransferase) and the expression of acetylated histone H3 were both decreased. After the blood glucose of the rats was restored to normal level, the above changes were irreversible, indicating that DR development is significantly associated with H3 acetylation, and the latter may participate in the formation of “metabolic memory” [24]. Based on the above results, we speculate that in retinal cells, the H3 deacetylation may downregulate the genes maintaining the redox state and inhibiting cell apoptosis, thereby resulting in the progression of DR.

Oxidative stress also plays a major role in regulating hyperglycemia-dependent histone deacetylation, which is speculated to be a pathogenic factor for the development of diabetic microvascular complications [25, 26]. The reactive oxygen species (ROS) could increase the activity of HDAC while decreasing that of HAT, thereby inhibiting histone acetylation. The mitochondrial dysfunction in the retina could cause apoptosis of endothelial cells and further lead to the development of DR [8]. The lysine-specific demethylase 1 (LSD1) is ascribed as the underlying factor, primarily functioning by decreasing the level of histone H3 dimethyl lysine 9 (H3K9me2) in the promoter region of MMP-9 and increasing the levels of acetyl H3K9 (Ac-H3K9) and p65 [27]. SOD catalyzes the disproportionation of superoxide radicals into oxygen or hydrogen peroxide, thereby aiding the cells in the removal of superoxide molecules. Since hyperglycemia inhibits the protective effect of Mn-SOD on retinal mitochondria, it also accelerates the apoptosis of endovascular cells and induces the development of DR in STZ-induced diabetic rats [28, 29]. In addition, histone modification within the *SOD2* gene is another crucial factor causing DR; significantly increased H3K9 methylation and H4K20 acetylation was observed in the promoter and enhancer regions of SOD2, respectively [30]. The lysine in different histones could be modified, and these modifications would result in various alterations. Under hyperglycemia, LSD1 could be activated, which reduces H3K4 acetylation in the *SOD2* gene, and ultimately induces the development and progression of DR.

### miRNA and DR

The blood retinal barrier (BRB) breakdown and neovascularization are the basic pathogenic characteristics of DR [31]; non-coding RNA, especially miRNA, could regulate the expression of genes involved in DR development, thereby complicating the sophisticated epigenetic modifications of DR.

The blood retinal barrier breakdown is the hallmark of DR [32]. Several miRNAs have been shown to be involved in the pathogenesis of DR. miR-23b-3p regulates high-glucose-induced cellular metabolic memory in cultured human endothelium cells through an SIRT1-dependent signaling pathway [33]. In a diabetic rat model, miR-126 was found to play a potential role in the pathogenesis of DR, which might regulate the expressions of VEGF, Ang-1, and VCAM-1 [34].

Previous studies have shown that miRNA plays an important role in regulating retinal neovascularization [35]. In mice with ischemic retinopathy, microarray analysis identified seven miRNAs with increased expression (miRNA-106a, -146, -181, -199a, -214, -424, and -451) and three miRNAs with decreased expression (miRNA-31, -150, and -184) [36]. miRNA-31 and miRNA-184 are specifically expressed in non-vascular cornea and lens and are found in the retina at a relatively low level [37], suggesting that these miRNAs may inhibit neovascularization in the cornea, lens, and retinal tissues. In rats with ischemic retinopathy, injection of pre-miRNA-31, -150, and -184 downregulate the expression of vascular endothelial growth factor (VEGF), platelet-derived growth factor B (PDGF-B), and hypoxia inducible factor-1α (HIF-1α) [36]; however, the potential targets of these miRNAs have not yet been identified. Nevertheless, the study suggested that elevating the level of miRNA through intraocular injection may become a potential choice for the treatment of DR [38]. An in vitro experiment on human retinal endothelial cells (HRECs) found that high-glucose induction resulted in decreased level of miR-18b, which facilitated cell proliferation and VEGF expression. Notably, miR-18 mediates signal transduction via the activation of the IGF-1/IGFR-1 signaling pathway [39] (Table 2).

**Table 2:** miRNA and diabetic retinopathy

Refs	Study type	Identified miRNA
Graefes Arch Clin Exp Ophthalmol [40]	Clinical study (serum & vitreous)	miR-15a, miR-320b, miR-93, miR-423-5p, miR-29a, miR-320a
Diabetes [23]	Clinical study (serum)	miR-27b, miR-454, miR-28-3p, miR-122, miR-320a, miR-125b, miR-221
Sci Rep [60]	Clinical study (serum)	miR-125b, miR-221, miR-132, miR-100, miR-376a
IOVS Nuria [41]	Clinical study (E/PCs)	miR-221, miR-126, miR-222
Cell Physiol Biochem [42]	Clinical study (serum)	miR-21, miR-181c, miR-1179
FEBS Lett [43]	Invitro/animal OIR mice study Retina	miR-184
BRBC [44]	Invitro/animal study Retina	miR-192-5P, miR-335
Diabetologia [34]	Invitro/animal diabetic rats (E/PCs)	miR-195
Circ Res [45]	Invitro/animal (STZ diabetic rat/db/db mice) Retina/(E/PCs)	IncRNA-myocardial infarction-associated transcript (MIAT)and miR—150
Int J Med Sci [46]	Invitro/animal STZ diabetic rat study Retina	miR-126
Mol Vis [47]	Invitro/animal STZ diabetic rat study Retina	miR-29b
IOVS [48]	Invitro/animal Akita mouse/(E/PCs)	miR-146,miR-200b
IOVS [49]	Invitro/animal Retina/(E/PCs)	miR-200b
IOVS [50]	Invitro/animal Akita and wild-type (WT) mice study Retina	miR-200b
Diabetes [51]	Invitro/animal (STZ diabetic rat) study Retina	miR-200b
Molecular Therapy [52]	Animal study (mice with oxygen-induced ischemic retinopathy) retina	miR-106a,-146,-181,-199a,-214,-424, -451 miR-31, miR-150, miR-184
The International Journal of Biochemistry & Cell Biology [32]	Invitro study In vitro cell proliferation analyses	MiR-18b

### Circular RNA and DR

**Fig. 1:**
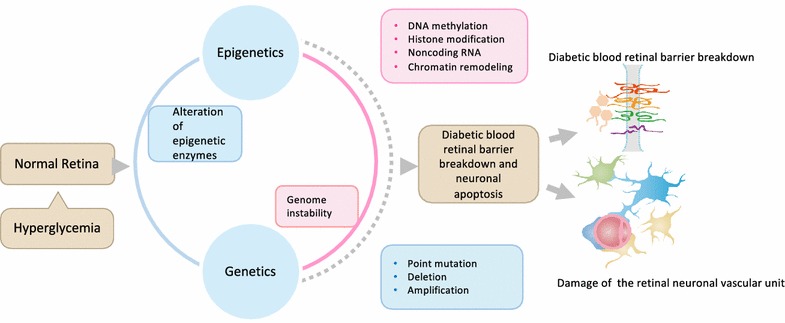
The role of genetics and epigenetics in the pathogenesis of diabetic retinopathy. Hyperglycemia induced breakdown of the blood retinal barrier (BRB) damage of the retinal neuronal vascular unit are the early pathogenic characteristics of DR. This figure illustrates the role of genetics (point mutation, deletion, amplification) and epigenetics (DNA methylation, histone modification, noncoding RNA, Chromatin remodeling) downstream of hyperglycemia in leading to diabetic retinopathy

Contrasting to linear RNA, the 3′ and 5′ in circular RNA, are joined and renamed circRNA. The circular RNA has been predominantly found in the cytoplasm and is usually composed of 1–5 exons, approximately 100 nucleotides in length with thousands of members in mammalian cells. Although these circular RNAs are more stable than the linear RNAs [53], their functions are yet to be clarified. Some of the potential roles might include: (1) evolutionary conservation of circularization mechanisms and signals and interactions with miR-7 [54–56], miRNA 26-28 due to its sponge capacity, as well as binding to RNA-binding proteins and initiating the in vivo protein production from the start codon [57]. (2) Regulation of mRNA in the cell through limited base pairing. (3) The physiological role of circular RNAs may be related to senescence, aging, and cell death [58]. (4) Circular RNAs have been shown to have a protective effect on colorectal cancer through downregulation of cir-ITCH expression [59]. (5) A study has shown that the circular RNA, Cdr1, via miR-7 and its targets, regulates insulin transcription and secretion in islet cells in vitro. Nonetheless, the functions of circular RNAs remain uncertain in the pathogenesis of DR. The role of genetics and epigenetics in the pathogenesis of diabetic retinopathy is summarized in Fig. 1.

#### Reversibility of epigenetic modifications and development of new molecular targets

The epigenetic modifications can precede disease pathology and serve as biomarkers to predict the development or progression of diseases. Furthermore, the main characteristics of epigenetic modification are also reversible, rendering them as promising therapeutic candidates.

As discussed above, histone acetylation resulted in the acceleration of DR. Garcinol, an inhibitor of the histone acetyltransferases [60], can prevent the epigenetic alterations that are implicated in the metabolic memory in DR. Histone deacetylase/DNA methylation inhibitors have been approved by the USA FDA and have been successfully applied clinically (ref). MiRNAs have also gained attention as potential therapeutic targets.

A novel gene therapeutic technique that efficiently inhibits the early pathologies in DR has been established: the epigenetic silencing of thioredoxin-interacting protein (TXNIP) in the diabetic retina [61]. TXNIP is induced early by hyperglycemia (in vitro) and diabetes (in vivo diabetic retina). TXNIP plays a central role in promoting neurovascular dysfunction in DR, and its epigenetic silencing prevents the progression of DR [61, 62]. This technique consists of injecting the retina of STZ diabetic rats with small interfering RNA (siRNA). These siRNAs are targeted to the TXNIP promoter and coupled via electrostatic bonds (not covalent) with cell penetrating peptides (CPP) containing a nuclear localization signal, in order to target the delivery into the cell nucleus and the TXNIP gene regulatory region [61]. Recent data show that miRNA plays a role in DR progression; thus, this technique may be used to restore the expression of specific miRNA in late DR by coupling with CPP.

Several possibilities for epigenetic treatment of DR have been studied. One approach is to inhibit the methylation of SOD2 and MMP-9. The DNMT inhibitors, 5-azacytidine, and 5-aza-20-deoxycytidine, have both been approved by the FDA for the treatment of other conditions. Further studies have examined the effects of those compounds on DR; wherein they seem to inhibit the methylation patterns with some success at reducing the symptoms. The DNA methylation inhibitor, Zebularine, has also been studied, although results are currently inconclusive. A second approach is to reduce the miRNAs observed at elevated levels in patients with DR, although the exact role of those miRNAs is yet unclear. The histone acetyltransferase (HAT) inhibitors, epigallocatechin-3-gallate, vorinostat, and romidepsin, have also been extensively studied albeit with limited success [63]. The possibility of using siRNAs to target the miRNAs mentioned above has also been speculated; however, no methods are currently known. The method described in the present study is partially impeded by the difficulty involved in delivering the siRNAs to the affected tissues [63].

In summary, the impact of epigenetics in DR is an emerging area, reversibility of epigenetic modification could provide a new strategy for the prevention and treatment of DR.

## Abbreviations

BRB: the blood retinal barrier
CPP: cell penetrating peptides
DEM: diabetic macular edema
DM: diabetes mellitus
DNMT: methyltransferase
DR: diabetic retinopathy
FDA: food and drug administration
HAT: histone acetyltransferase
HDAC: histone deacetylases
H3K9me2: H3 dimethyl lysine 9
HIF-1α: hypoxia inducible factor-1α
HLM: histone lysine methylation
HRECs: human retinal endothelial cells
LSD1: lysine-specific demethylase 1
miRNA: microRNA
MMP9: matrix metalloproteinase 9
NK: natural killer
PDGF-B: platelet-derived growth factor B
POLG1: polymerase γ-1
ROS: reactive oxygen species
SAM: S-adenosylmethionine
siRNA: small interfering RNA
SOD: superoxide dismutase
STZ: streptozotocin
TXNIP: thioredoxin
VEGF: vascular endothelial growth factor
